# Utilizing Dimensions of Trust to Communicate with Consumers About the Science Behind Food

**DOI:** 10.3390/foods14101674

**Published:** 2025-05-09

**Authors:** Alexa J. Lamm, Kevan W. Lamm, Allison R. Byrd, Nicholas Gabler, Catherine E. Sanders, Michael S. Retallick

**Affiliations:** 1 Department of Agricultural Leadership, Education & Communication, University of Georgia, Athens, GA 30606, USA; kl@uga.edu (K.W.L.); allisonbyrd@uga.edu (A.R.B.); 2 Department of Animal Science, Iowa State University, Ames, IA 50011, USA; ngabler@iastate.edu; 3 Department of Agricultural and Human Sciences, North Carolina State University, Raleigh, NC 27695, USA; 4 Department of Agricultural Education and Studies, Iowa State University, Ames, IA 50011, USA; msr@iastate.edu

**Keywords:** trust, consumer, food production, new food acceptance, cluster

## Abstract

Communicating the science behind food production to consumers is increasingly complex due to the proliferation of food innovations, information overload, and the presence of misinformation. Trust plays a pivotal role in consumer perceptions of food safety and acceptance of new food technologies. This study explores consumers’ trust in food by segmenting audiences based on cognitive trust in science, affective trust in new foods, and dispositional trust in sources of food information. Using a survey of 1011 United States consumer respondents, cluster analysis identified five distinct trust segments: Lack Trust, Trusting, On the Fence, Trust New Food Not Science or Sources, and Trust Science not New Food. Results revealed significant demographic differences among the five segments, with age, education, political ideology, and dietary preferences influencing trust levels. Findings contribute to audience segmentation theory by demonstrating the coexistence of multiple trust dimensions and their impact on food-related decision-making. Practically, this study provides a framework for science communicators and policymakers to tailor messaging strategies that align with consumer trust profiles, ultimately fostering informed decision-making in the food system.

## 1. Introduction

Communicating with consumers about the science behind food production, food composition, and food’s role in health is increasingly challenging given the quickly growing diversity of innovations available in the food production space ranging from the use of artificial intelligence (AI) in recipe ideation and food making [1] to genetic technology as a part of the food production system [2] and the acceptance of 3D-printed food [3]. However, convincing consumers to try new foods is not a novel dilemma. Humans have historically been cautiously interested in trying unfamiliar foods due to the risk associated with eating something new and potentially dangerous [4]. The phenomenon of food neophobia or “a psychological trait characterized by a general reluctance to eat new/unusual foods” (p. 708) [4] has been studied across multiple cultures over many decades because of food neophobia’s potential to negatively influence diets in the modern food system in which most food is typically safe. Research has revealed food neophobia generally tends to decrease as people age but increase once they become elderly [4], while social elements such as socioeconomic status, education level, political ideology, and being exposed to new foods can influence consumers’ levels of food neophobia [5,6]. Therefore, communicators must consider the psychographic and demographic characteristics that influence willingness to try new foods when examining how consumers think about the science behind the food they eat.

Consumers are also skeptical of food and the safety thereof due to various food safety events in an increasingly globalized food system [7,8]. The globalized food system offers new, nutritionally diverse, and beneficial foods, and consumers must rely on a variety of sources for food quality, safety, and nutrition information, all of which affect their trust in the broader food system [8]. However, finding reliable information sources is increasingly complex thanks in part to food myths—beliefs perceived as truth by consumers about the nutrition of certain foods that are incorrect or directly contradict empirical findings—a global problem affecting human nutrition and consumption of certain foods [9]. Food myths and inaccurate nutrition information have been perpetuated by the spread of inaccurate and low-quality information by online influencers across various internet platforms [10], prominent sources of nutrition information for many young consumers who do not possess the skills to determine the scientific accuracy of online content [11]. Social media use can serve as a powerful determinant of attitudes toward new food product information and combined with trust, influence consumers to adopt new food products, such as plant-based meats [12]. Therefore, there is a need for an empirical examination of the various factors influential in shaping perceptions of food information sources in the current food climate.

Previous research into food information sources has recognized that audiences trust university scientists, farmers, and environmental organizations over governmental entities or food manufacturers when seeking information about certain food issues such as genetically modified (GM) food [13]. Household income levels have been reported to have a significant relationship with trust in university scientists, while age was inversely related to how much respondents trusted in governmental agencies for information concerning GM food [13]. In a five-country European study, consumers also found farmers more trustworthy than governmental authorities or food manufacturers [14]. Findings also revealed individuals’ levels of trust in “food chain actors…[was] chiefly determined by their beliefs about the competence, care, and openness of these actors” (p. 11) [14]. This indicates that nuanced perceptions of different players in the food system (such as farmers, regulators, or manufacturers) influence how consumers choose to trust the overall food system. A study by Robinson et al. (2020) [15] indicated that a difference in consumers’ most used communication source, gender, and engagement in agriculture all influenced consumers’ trust in farmers, food retailers, food processing, and regulations upholding food safety. Respondents personally engaged with agriculture were more likely to trust food processing than those who were not, while respondents who used television as their top information source were more likely to trust in food processing than those who preferred social media or print media [15]. Women were also more likely than men to have lower levels of trust in both food processing and food safety regulation [15]. Given the differences in trust displayed by audiences with various personal and demographic characteristics in previous research, a targeted look at the characteristics which influence trust in sources of food information is necessary for the current food production climate.

While communicating with consumers about the science behind their food is a daunting task, the burden has recently been exacerbated by a decline in trust in scientists [16]. Since the onset of the COVID-19 pandemic in 2020, public trust in scientists has suffered a blow in the United States (U.S.), especially amongst political conservatives [16]. Additionally, public entities tend to distrust governmental organizations, including those responsible for setting and enforcing policies associated with food safety, such as the U.S. Department of Agriculture and the Food and Drug Administration [13,14,15]. Therefore, understanding public trust in science is a matter of global importance [17], as is understanding trust in the food system [14]. Investigating the nuances of consumer trust can not only shape communication with consumers but influence policy decisions in the broader food system [14]. Therefore, exploring the factors that influence trust in the food system, information sources, and trust in science is of paramount importance in an increasingly divided global society.

## 2. Literature Review

The Situational Theory of Publics [18] provides a foundation for segmenting publics based on problem recognition, constraint recognition, and level of involvement with the subject matter being discussed. Publics, or segmented audiences, identified within the larger group can then be categorized as latent, aware, or active based on their level of engagement with a specific topic [19], in this case, food consumption. Strategic messages can be developed which target the expansion of knowledge, influence attitudes, and promote behavior change for specific audiences [20], essentially clustering people into socially motivated groups rather than targeting a larger, more diversified population [21]. Using demographics and psychographics to “segment by lifestyle or personality traits or characteristics” (p. 46) [22] has been successfully implemented to improve food-focused communication efforts [23]. However, targeted food messaging strategies can accidentally polarize audiences [20,23] if the information presented is perceived as being manipulated [24]. Therefore, fostering a comprehensive understanding of how and why specific audiences trust or do not trust information about food production practices may be a crucial step in establishing effective communication when it comes to sharing scientific information related to food production practices [23,25,26].

Trust is defined as an “assured reliance on the character, ability, strength, or truth of someone or something” or “one in which confidence is placed” (P1) [27]. Based on this definition, cognitive trust, or the objective truth consumers expect from scientists to ensure food is safe to eat, is imperative. It has been claimed that scientific knowledge is perceived by a consumer as trustworthy when it is (1) produced through objective processes, (2) reliably replicated across multiple experimental contexts, or (3) the result of a critical discussion between diverse members of the scientific community [28].

Previous research has found that consumer trust in the food system is influenced largely at the product level through labeling or indirectly through actors such as producers, processors, retailers, government agencies, advocacy groups, and the mass media [8]. This is an indicator that knowledge shared by the scientists behind food production (e.g., through food labeling) is not the only way trust is established between consumers and the food system’s actors. Philosophical arguments have been made that the broadly accepted definition of trust is oversimplified, as trust stretches beyond mere reliance [29] due to the possibility of betrayal [30]. The possibility of being betrayed indicates that affective trust, or the role of shared values, interests, and social perspectives [31] may impact how consumers think and feel about the science behind food production practices. As a result, trust is not purely built on trust in the scientific process but in the perceived level of shared values with the groups or individuals creating and sharing scientific information.

Many marketing campaigns, regardless of the product being sold, have depended upon these two aspects of trust (cognitive and affective) to build consumer trust [32,33,34]. However, dispositional trust is often ignored despite being found as a predictor of consumer trust across product dimensions. Other researchers have found consumers “perceived a trusting or distrustful outlook as a personality characteristic” (p. 350) [35]. Previous research has also found individuals express dispositional trust as a natural trusting or cynical personality trait that impacts their innate willingness to trust any individual or organization across contexts [35] and should be taken into consideration when segmenting audiences. Therefore, by identifying specific segments of consumers based on their levels of cognitive, affective, and dispositional trust, effective science communication messages can be developed to help people use science when making decisions about the food they eat and the food production methods they support.

## 3. Purpose and Research Objectives

The purpose of the study was to explore the characteristics of different groups of U.S. consumers based on cognitive, affective, and dispositional trust. The research was guided by the following research objectives:

(1) Identify distinct clusters of U.S. consumers based on their level of cognitive trust in science, affective trust in new food, and dispositional trust in sources of food information.
(2) Describe demographic characteristics of U.S. consumers based on their membership in distinct trust clusters.

## 4. Methods

The quantitative survey instrument used for this study was part of a larger research endeavor to investigate public perceptions of pork consumption so that communication efforts can be tailored to specific needs.

### 4.1. Sample and Data Collection

The target population was U.S. consumers 18 years of age or older. Data were collected from a representative sample using Qualtrics, an online survey platform, in June 2024. Non-probability opt-in sampling was used to collect the data [36]. Public opinion research uses non-probability opt-in sampling to make population estimates [37]. Results have indicated using this sampling method has obtained responses equal to, and sometimes better than, those obtained when using probability sampling methods [36]. Data collection resulted in 1011 consumers’ responses to the survey, indicating an adequate sample size for data analysis [38]. Qualtrics compensated respondents according to their standard protocols. The use of the Internet to recruit respondents does have limitations. Internet access and individuals inclined to opt in to incentivized panels can introduce sampling bias. *A priori* quotas were established prior to data collection based on gender, age, race/ethnicity, and geographic location to ensure the sample collected was representative of the current U.S. population at the time of data collection [37]. *Post hoc* weighting techniques were also applied prior to data analysis to ensure that responses adequately represented the U.S. population [36].

### 4.2. Instrumentation

Data were collected using demographic and Likert-type scale questions using an online survey. Cognitive trust was measured using the Public Trust in Science Scale (PuTS) [39]. The five-item scale assesses cognitive trust in scientists based on perceptions of their credibility, transparency, ethical behavior, and adherence to rigorous standards in their professional activities [39]. The instrument asked respondents to indicate their level of agreement or disagreement with five statements using a five-point Likert-type scale (1 = Strongly Disagree; 2 = Disagree; 3 = Neither Agree nor Disagree; 4 = Agree; 5 = Strongly Agree). Although the items are not explicitly food-specific, this generalized measure of cognitive trust is widely recognized as a foundational determinant of public attitudes and acceptance towards scientific applications broadly, including those in food science and biotechnology [8,40,41]. Scale reliability was calculated *post hoc* (α = 0.86).

Affective trust in new food was measured using a four-item scale adapted from a previous study [42]. The instrument asked respondents to indicate their level of agreement or disagreement with each statement using a five-point Likert-type scale (1 = Strongly Disagree; 2 = Disagree; 3 = Neither Agree nor Disagree; 4 = Agree; 5 = Strongly Agree). The four statements were all positively framed. The mean score of the responses to the four items was used to create an overall scale for affective trust in new food. Scale reliability was calculated *post hoc* (α = 0.76).

Dispositional trust was conceptualized as a generalized propensity to regard a wide array of food information sources as credible, consistent with trust frameworks that depict dispositional (trait–level) trust as a cross-situational tendency rather than a source-specific judgement [43,44,45]. Specifically, dispositional trust in sources of food information was measured using a 7-item researcher-adapted scale adapted [46]. The instrument requested respondents indicate how trustworthy or untrustworthy they found each source regarding delivering accurate information about the food they eat/feed their family using a five-point Likert-type scale (1 = Very Untrustworthy; 2 = Somewhat Untrustworthy; 3 = Neither Trustworthy nor Untrustworthy; 4 = Somewhat Trustworthy; 5 = Very Trustworthy). To establish dimensionality, the sample (*N* = 1011) was split randomly following scale-development guidance for cross-validation [47]. An exploratory factor analysis on the calibration subsample (*n* = 510) using principal-axis factoring produced a single-factor solution (eigenvalue = 2.61) on which all items loaded ≥ 0.50 and explained 37% of the variance. Confirmatory factor analysis on the validation subsample (n = 501) supported this one-factor model (*χ^2^* = 119.7, *df* = 14; CFI = 0.90; SRMR = 0.056; RMSEA = 0.123). Standardized loadings ranged from 0.46 to 0.77. Composite reliability (CR = 0.81) exceeded the 0.70 benchmark, while the average variance extracted (AVE = 0.39) was marginally below the conventional 0.50 cut-off. AVE values ≥ 0.40 are acceptable when CR is >0.60, indicating adequate convergent validity for exploratory work [48]. Given the lack of conceptual overlap with the other study constructs, discriminant validity was deemed satisfactory. A mean score across the seven items was therefore retained to represent respondents’ dispositional trust in sources of food information.

The instrument was reviewed for face and construct validity by a panel of experts in survey design, animal science, science communication, and agricultural education prior to pilot testing. The research design was then approved by the University of Georgia Institutional Review Board (IRB #00008098) and pilot tested (*n* = 50) with individuals who were representative of the sample. The instrument was not changed following the pilot test given the accuracy of the measurement scales. To assess the potential presence of common method bias (CMB), a common latent factor (CLF) analysis was used following the recommended procedures [49,50]. This approach involved estimating two confirmatory factor analysis (CFA) models: a baseline model specifying the hypothesized three-factor structure (Cognitive Trust, Affective Trust, and Dispositional Trust) and a second model that included an additional CLF to account for shared method variance. In the baseline model, fit indices indicated acceptable model fit [51], with a standardized root mean squared residual (SRMR) of 0.054, comparative fit index (CFI) of 0.877, Tucker–Lewis index (TLI) of 0.854, and a root mean square error of approximation (RMSEA) of 0.085. The Akaike information criterion (AIC) and Bayesian information criterion (BIC) were 41,206.45 and 41,457.30, respectively. The model was then re-estimated, including a CLF, which was constrained to a variance of 1.0 for model identification and allowed to load on all observed indicators. The CLF model produced an improved SRMR of 0.039 and a perfect CFI of 1.000, with a coefficient of determination (CD) of 0.997. As expected in CLF models, RMSEA and model chi-square were not computed due to non-identification [49]. Model complexity led to increases in both the AIC (41,294.60) and BIC (41,609.39). Although the AIC and BIC values increased slightly with the inclusion of the CLF, the differences (ΔAIC = 88.15; ΔBIC = 152.09) remained within a range suggesting only weak to moderate evidence of improved model fit [52,53]. This supported the conclusion that common method variance did not substantially bias the model. Detailed item descriptions, reliability, and sources of survey items can be seen in Table 1.

### 4.3. Demographics of Respondents

Respondents ranged in age from 19 to 90 (*M* = 50.68, *SD* = 10.09). Approximately three fourths of the respondents were White (74.9%), nearly half had at least a two-year college degree (48.6%), and almost all had a total family (household) income (before taxes) of less than USD 149,999 (92.6%). Most respondents were either single or married (68.8%), and 28.3% had children under the age of 18 currently living in their home. Only 11.3% of the respondents reported having a special diet (vegetarian, pescatarian, vegan, or paleo). Full demographic profiles prior to weighting can be seen in Table 2.

### 4.4. Data Analysis

Two-step hierarchical and K-means cluster analysis were used to identify distinct groups of U.S. consumers based on their level cognitive trust in science, affective trust in new food, and dispositional trust in sources of food information. Cluster analyses are data reduction techniques that take large datasets and organize responses into smaller, maximally dissimilar groups, also known as clusters, based on response patterns [54,55,56,57]. Social science studies have used cluster analysis to determine audience segments in agricultural and environmental contexts broadly [54,58,59], assisting in the formation of more informed and effective science communication. In theory, individuals exhibiting similar needs from educational experiences and communication materials in a study population will be represented by the segments of clusters [56,58].

Cluster analysis, using the input variables, was run on 1011 cases using SPSS 29 (Chicago, IL, USA). First, correlations between cognitive trust in science, affective trust in new food, and dispositional trust in sources of information related to the food they eat were examined to ensure multicollinearity was not an issue. All correlations were below 0.52 and thus deemed acceptable based on the literature [60]. No outliers were found within the dataset, and therefore, removal was not needed. Variables were equally distributed. Therefore, there was not a need to center, modify, or standardize prior to analysis.

**Figure 1:**
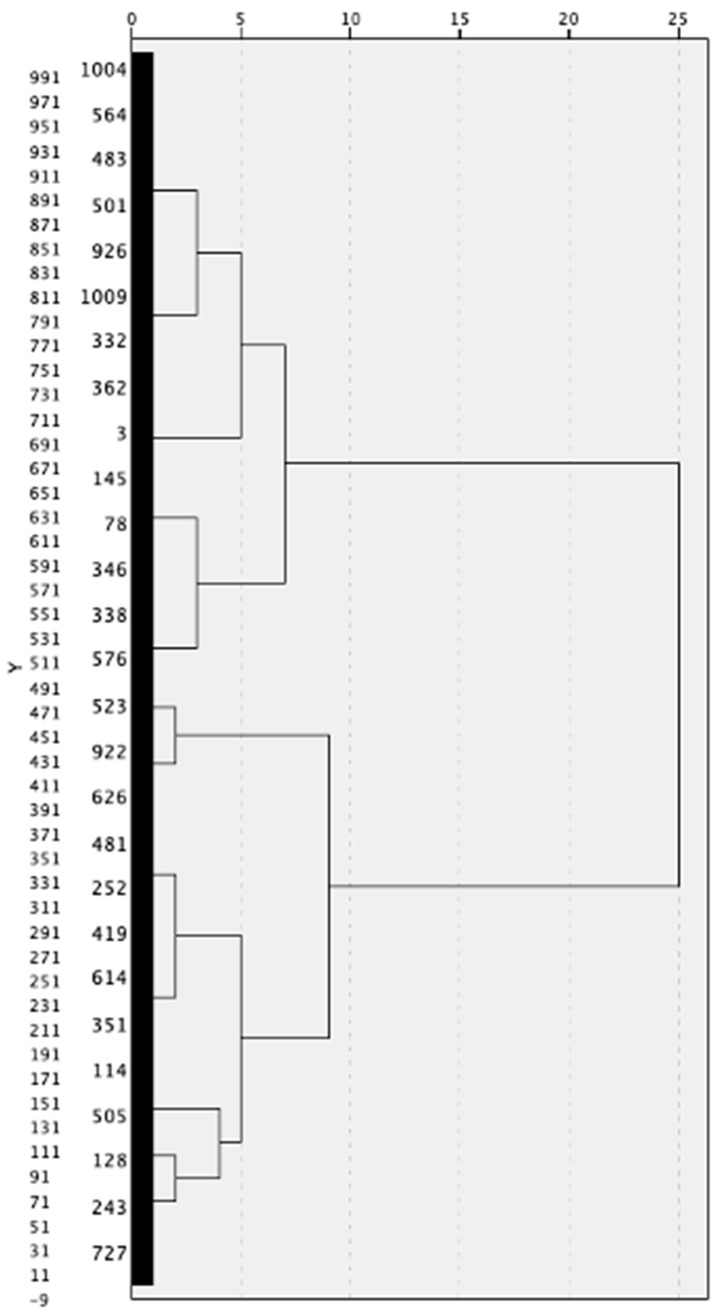
Dendrogram output using Ward linkage to determine number of clusters.

A hierarchical cluster analysis was conducted using Ward’s method specifying squared Euclidean distance to determine the number of subgroups that would achieve maximum dissimilarity between resulting subgroups [57]. Ward’s method is commonly used when the number of clusters needs to be determined [55]. The cutoff value of 15 and the largest distance between clusters (vertical lines) in the dendrogram (Figure 1) were used to determine an appropriate number of subgroups [57]. Following the hierarchical visual appraisal, the agglomeration schedule was analyzed to empirically confirm the appropriate number of emergent clusters. The agglomeration schedule from the hierarchical cluster analysis revealed a marked increase in fusion coefficients between the 5-cluster and 4-cluster solutions (from 844.83 to 944.54; Δ = 99.71), indicating a substantial loss of between-cluster heterogeneity if further clusters were merged. This type of “elbow” in the agglomeration coefficients is a commonly recommended criterion for determining the optimal number of clusters [48,57,61]. The magnitude of the increase at this stage represented the largest jump in the final stages of clustering, thus supporting the retention of a five-cluster model. Each final cluster consisted of mutually exclusive and previously unmerged groupings, ensuring methodological rigor and coherence. These initial results from Ward’s method were then validated and refined using K-means clustering, as described below, consistent with recommendations in the literature [48,61,62].

K-means clustering, which allows the researcher to set the number of clusters for the analysis, was conducted using the results of the Ward’s method analysis to partition the dataset into appropriate subgroups (e.g., 5, 10). The maximum iterations were adjusted from 10 to 99 to avoid any issues with early convergence in the solution. Convergence was achieved in 20 iterations. To assess the stability of the five-cluster solution across methods, a Cohen’s kappa coefficient was calculated to compare cluster assignments generated by Ward’s method and K-means clustering. The analysis yielded a kappa value of 0.519 (*p* < 0.001), indicating moderate but statistically significant agreement between the two methods. This result supports the validity and internal consistency of the derived cluster structure, while also acknowledging some variation in case classification between approaches [63]. Based on theoretical coherence and interpretability, the five-cluster K-means solution was retained for additional analysis. To assess the practical significance of differences between clusters across the trust dimensions, partial eta-squared (η^2^) values were calculated as part of the ANOVA procedure in SPSS. Partial eta squared represents the proportion of variance in the dependent variable explained by the clustering variable, controlling for other sources of variation. Following guidelines from the literature [64], η^2^ values of approximately 0.01, 0.06, and 0.14 were interpreted as small, medium, and large effects, respectively. Partial eta-squared (η^2^) values for dispositional trust (η^2^ = 0.572), cognitive trust (η^2^ = 0.543), and affective trust (η^2^ = 0.665) indicated large effects. These results suggested that the observed differences between clusters were not only statistically significant but also substantively meaningful [65]. Additionally, associations between cluster membership and demographic characteristics were also assessed using Cramer’s V. Based on guidelines in the literature [66], V values of approximately 0.10, 0.30, and 0.50 were interpreted as small, medium, and large effects, respectively. The findings indicated a small effect size for sex (V = 0.131), a small/borderline effect size for education level (V = 0.088), and a small-to-moderate effect size for political ideology (V = 0.161) [66].

Individual cases were assigned to five clusters. Analysis of variance (ANOVA) was then used to determine the magnitude of difference between the variables of interest, age, and cluster membership. Chi-square analyses were used to determine significant differences between the cluster groups’ categorical demographics (e.g., sex, education level, family income).

## 5. Results

Five distinct audience segments emerged through the cluster analysis (see Table 3). The audience segments (identified as clusters in the analysis) were distinctively different in their level of cognitive trust in science (*F* = 298.96, *p* < 0.001, *η*^2^ = 0.54), affective trust in new food (*F* = 499.59, *p* < 0.001, *η*^2^ = 0.67), and dispositional trust in sources of information related to the food they eat (*F* = 335.51, *p* < 0.001, *η*^2^ = 0.57). Subsequently, names were selected to represent the identified audience segments: Lack Trust, lowest levels of trust in all three domains (*n* = 108); Trusting, consistently high trust across all domains (*n* = 174); On the Fence, moderate trust levels across all domains (*n* = 272); Trust New Food Not Science or Sources, more open to new food technologies but expressed lower trust in scientific institutions and communicators (*n* = 202); and Trust Science Not New Food, high levels of trust in scientific sources and institutions but comparatively lower trust in novel food technologies (*n* = 255). The Trusting segment, which exhibited the highest levels of trust in all three areas, was the youngest group, with an average age of 46.26. Differences in levels of trust across the five segments can be seen in Table 3.

*Post hoc* comparisons using the Games–Howell (equal variances not assumed) procedure revealed that all five clusters differed significantly from one another in cognitive trust scores (*p* < 0.001). The Trusting cluster reported the highest cognitive trust, followed by Trust Science Not New Food, On the Fence, Trust New Food Not Science, and Lack Trust, which reported the lowest. Similar statistically significant differences in affective trust scores across all five clusters (*p* < 0.001) were also observed. *Post hoc* comparisons revealed that most clusters differed significantly in dispositional trust scores (*p* < 0.05), with two exceptions. No significant differences were found between the Trust Science Not New Food and On the Fence clusters (*p* = 0.003) nor between the Trust New Food Not Science and Lack Trust clusters (*p* = 0.023), indicating some overlapping patterns of general trust in information sources. The Trusting cluster reported the highest dispositional trust, while the Trust New Food and Lack Trust clusters reported the lowest.

Significant differences were identified between the five segments when demographics were analyzed. More female respondents were in the Lack Trust (64.8%) or Trust Science Not New Food (61.6%) segments than their male counterparts (*X*^2^ = 17.29, *p* < 0.01). African-American respondents were more likely to be in the Trust Science Not New Food segment (21.5%) than any other segment. Similarly, Asian and American Indian/Alaskan Native respondents were more likely to be in the Trust New Food Not Science or Sources segment than any other segment. Conversely, respondents indicating Hispanic ethnicity were more likely to be in the Trusting category, and statistically significant differences were found across all segments (*X*^2^ = 11.01, *p* < 0.01).

Respondents reporting a higher level of education were more inclined to be in the Trusting segment than those reporting a lower level of education. Differences in education level across all segments were statistically significant (*X*^2^ = 30.98, *p* < 0.05). Marital status also differed significantly across the five segments (*X*^2^ = 37.27, *p* < 0.01). Respondents who reported that they were single were most present in the Trusting segment, while those reporting that they were married were most likely to be found in the Trust New Food Not Science or Sources segment. In addition, respondents reporting that they were divorced were most likely in the Trust Science Not New Food segment, while those who were widowed appeared most often in the Lack Trust segment. However, respondents who did not have any children under the age of 18 currently living in the home were least likely to be in the Trusting segment, while respondents with children were more likely to be in the Trusting segment than the others (*X*^2^ = 40.58, *p* < 0.001).

Reported family income also had statistically significant differences between the five segments (*X*^2^ = 47.25, *p* < 0.001). Respondents who reported they had a family income of USD 24,999 or less were more likely to be in the Lack Trust segment, whereas those with a family income of USD 75,000 or greater appeared more often in the Trusting segment. Political ideology also differed between segments (*X*^2^ = 105.37, *p* < 0.001), with members of the Trusting category most likely being very liberal, and very conservative respondents most likely being in the Lack Trust segment.

Special diets did not have statistically significant differences observed between clusters. Details regarding distributions of characteristics within each of the five segments can be viewed in Table 4.

While many results were statistically significant, Cramer’s V was calculated to evaluate effect sizes given the large sample size (N = 1011). The effect sizes for most variables were small (Cramer’s V ranging from 0.09 to 0.13), suggesting modest practical differences. Political ideology exhibited the strongest association with trust segment alignment (Cramer’s V = 0.16), indicating a moderate effect. These findings highlight that while demographic characteristics differ across trust clusters, the magnitude of these differences should be interpreted cautiously considering effect sizes.

## 6. Discussion

The results of the present study examined consumer perceptions and tendencies as it relates to trust and food consumption, with a particular focus on pork consumption. As the literature has previously established, there are numerous considerations that relate to consumer decision-making within the food system [1,2,3]. The results indicated there may be underlying trust-related factors which influence food-related decisions.

### 6.1. Contributions to Theory

From a theoretical perspective, there are four primary contributions which the present study makes to the existing literature. First, the results indicated that there is the potential for different modes of trust to exist simultaneously within an individual, in terms of food consumption preferences. Second, the use of clustering techniques may provide a novel way in which to examine sociographic variables of interest and their alignment with demographic characteristics. Third, demographic groups were observed to have differing levels of trust, indicating the potential for further audience segmentation and an ability to target food communication efforts. Fourth, the resulting clusters and associated frequency counts provide a heuristic understanding of potential population trust distribution.

The results indicated that the use of independent trust conceptualizations may be insightful in relation to trust and food consumption preferences. For example, the results indicated that the dispositional, affective, and cognitive trust conceptualizations may have some overlap; however, the conceptualizations appear to be mutually exclusive. Specifically, individuals with high levels of dispositional trust did not necessarily express high levels of affective or cognitive trust. This result is observable as it relates to the different clusters which emerged through the analysis process. An associated implication would be to consider trust at varying levels of both liminal and subliminal mental processing amongst potential audience and consumer segments. This finding aligns with previous research which has found that the development of marketing campaigns based on building both cognitive and affective trust between consumers and a brand are most effective [32,34]. Based on these findings, a recommendation would be to replicate the study in different food system-related scientific areas of inquiry such as the use of genetic engineering, water management of crops, and reducing plastic use on farms. Further exploration across the food system may help to further elucidate the nature of the relationship between dispositional, affective, and cognitive trust conceptualizations and consumer trust in the science behind food production. An additional recommendation is to potentially use alternative measures to represent dispositional trust. Although the composite of seven items used within the current study loaded on a single factor, a more robust latent variable with appropriate fit for purpose characteristics may provide more robust and replicable results. A similar recommendation applies to the PuTS scale and serving as a proxy for cognitive trust. Alternative measures may provide a more robust representation of trust occurring as a cognitive process.

A second primary contribution to the theoretical literature emerging from the present study is the use of clustering techniques to examine and identify unique audience and consumer segments. From a praxis forward perspective, the emergent *trust clusters* may provide a novel foundation upon which to consider future food-system-related science communication and outreach efforts. The methodological approach used outcome variables of interest (in this case, the three conceptualizations of trust) which were then used to empirically classify individuals. Grounding the work in the situational theory of publics [18] and considering previous audience segmentation research [21,22,23], the present study used demographic characteristics of respondents as variables of interest to which one can align with the emergent clusters. However, this methodological approach may be appropriate and applicable using other independent variables of interest. For example, it may be insightful to use personality traits other than being cynical [35], such as introversion/extroversion, critical thinking style, information seeking preferences, or communication channel preferences, as independent variables which are then aligned to clusters. This would be consistent with recommendations associated with the situational theory of publics [18]. A further recommendation would be to examine whether additional psychographic variables, communication platform variable preferences, or alternative audience-related characteristics may provide even more nuanced insight as it relates to trust cluster composition associated with food.

The results of the present study also provide an empirically grounded foundation within the literature to begin the development of consistent trust cluster demographic characteristic identification. Specifically, the Trusting cluster had the lowest observed mean age at 46. The On the Fence and Trust Food Not Science or Sources clusters had similar ages associated. At the older end of the age continuum, the Lack Trust and Trust Science Not New Food clusters were associated with the highest observed ages. Statistically significant differences were observed in relation to sex, ethnicity, education, marital status, family income, children under the age of 18 currently living at home, and political ideology. As a standalone study, these results should not necessarily be over-interpreted; however, there is evidence to suggest that there may be underlying patterns in terms of trust and demographics, which warrants further investigation. A recommendation would be for future research to follow a similar methodological protocol and examine whether observations are replicable.

Lastly, the results provide a quantification of audience segment distributions amongst trust clusters. Specifically, the largest percentage of respondents, 26.9%, were in the On the Fence cluster. This would indicate that over one quarter of all potential consumers are not necessarily decided as it relates to their trust in the food system. The Trusting cluster had 17.2% of all respondents. On the contrary, the Lack of Trust cluster had 10.7% of respondents. The Trusting and Lack of Trust clusters may be conceptualized as residing at opposite ends of the trust continuum. Within the trust continuum, two additional trust clusters emerged: Trust New Food Not Science or Sources and Trust Science Not New Food. These two clusters may be conceptualized as similar in that both have trust limitations; however, the specific area of trust deficiency is different. The ratio of Trusting Science Not New Food versus Trusting New Food Not Science or Sources was approximately 1.26 to 1. Overall, these distributions provide an empirical foundation which should be further investigated and re-examined through replication. For example, geographically based analysis may also yield novel insights into whether respondent location may be a relevant factor in the emergence of trust cluster categorization.

### 6.2. Contributions to Practice

Findings from this study provide several contributions to practice. Using audience segments to better communicate science with potential consumers as it relates to trust in the food system was confirmed and aligned with previous research [23,25,26]. Specifically, there are three primary areas of contribution. First, the findings provide a heuristic guide where efforts may be most beneficially deployed. Second, the study identifies the demographic characteristics most strongly associated with specific trust clusters. Lastly, the findings show the use of trust clusters can provide a novel lexicon within which to engage consumers.

From an overall perspective, one of the primary contributions of this study is the identification of unique trust clusters. Using the observed results may provide guidance on the engagement of different segments of consumers. Based on finite resources available for engaging and educating consumers, the results indicated that focusing on differing levels of trust may be more effective than simply on specific demographic characteristics [21]. Specifically, the results indicated there may be a large segment of the population which already has a high degree of existing trust with the food system. Therefore, a recommendation would be to monitor this segment but to allocate resources elsewhere with the potential of enhancing trust in and amongst other audience segments.

Conversely, there is a noteworthy segment of the population which does not appear to have trust in general. Again, from an allocation of resources perspective, attempting to address multiple areas of trust—dispositional, affective, and cognitive—within this group may not result in a modification to trust overall and, therefore, may not be worth the effort. As such, the findings imply that the three remaining clusters—On the Fence, Trust New Food Not Science or Sources, and Trust Science Not New Food—are the most favorable clusters for direct engagement.

Two clusters, On the Fence and Trust Science Not New Food, represent over 50% of all respondents. Perhaps focusing efforts on improving the overall affective trust in food with these two groups would be most advantageous. This investment may have an asymmetrical benefit relative to others based on the potential to elevate trust overall. A recommendation would be to partner with health professionals and dietitians to improve awareness of and trust in new foods amongst this targeted audience.

The second primary contribution from the study relates to the tactical deployment of efforts to engage specific demographic groups. The cluster analysis provided frequency-level and demographic-level distributions. Within the Trust Science Not New Food cluster, the demographic results indicated individuals within this cluster were more likely to be female, divorced (or single), have no children under 18 at home, and hold moderate or liberal political ideologies. Given these empirical observations, a recommendation would be to further analyze where an audience with these characteristics sources their news, establishes social norms, and develops cultural trends. By partnering with traditional and social media opinion leaders which this audience tends to follow; food communicators are more likely to improve awareness and acceptance of new foods by increasing affective trust.

Finally, the results also provided a novel set of descriptions for food audience segments. Specifically, the five named clusters may serve as a common set of descriptions for use within the food system. More narrowly defined audience segments allow for more precise engagement and interventions [22]. The emergent trust clusters are logically distributed across a continuum from non-trusting to trusting, with various dimensions of trust emerging between the two extremes. A recommendation is to continue implementing these descriptions within both the literature and practice to better describe specific aspects of trust within the food system. Like other cognition-based schemas, classifying trust across domain areas may provide increased description fidelity and associated intervention effectiveness.

### 6.3. Limitations

Although there are contributions to both theory and practice, there are several limitations which also must be acknowledged. First, as a non-probability opt-in sample, the results should not be extended beyond the respondents. Although care has been taken to ensure respondent representation based on census data, the findings should not be extrapolated to the broader population. Additionally, there are individuals in the U.S. who do not have access to the internet, and not everyone will respond to survey requests [37]. In addition, the entire survey was focused on meat and meat consumption. Therefore, certain individuals with specific dietary restrictions may have felt pressured to conform to their perceived norms and restrictions associated with new food selection [67].

Second, the establishment of the trust cluster concept should be recognized as novel and emergent. Further analysis and connection to theory would help to improve the concept and establish it more rigorously within the literature. Third, as noted previously, both the dispositional and cognitive trust measures used in the study may not provide the most robust representation of the underlying phenomenon of interest. Although statistical investigation indicated sufficiency, alternative measures may be warranted. Fourth, the study is focused on pork consumption; therefore, there is the potential that the emergent trust clusters are associated with this product, not with the food system more generally. Replicating the study using different food products of interest may be appropriate to determine generalizability of results. Lastly, as with all demographically based analysis, it is important to ensure results are not misinterpreted. General trends may be helpful in informing strategy and operational choices. However, a recommendation is to consider and respect everyone and their individual experiences.

## 7. Conclusions

Communicating with consumers about the food system and the science behind food production practices has traditionally followed consumer marketing and communication recommendations. However, given the visceral and proximal nature of the food system to the individual, this study proposed a novel representation of trust as core to future food science communication efforts. The emergence of trust clusters, and the empirical analysis of demographic trends amongst clusters, provides a robust set of quantitative data to inform future audience segmentation and food science communication efforts. As the global population continues to increase, efforts to better inform and communicate with the public about the science advancing the food system will be of paramount importance.

## Figures and Tables

**Table 1:** Survey items used to measure cognitive trust in science, affective trust in new food, and dispositional trust in sources of food information.

	Number of Items	α	Statements	Source
Cognitive trust in science ^a^	5	0.86	Scientists can be trusted because they are experienced experts in their particular topic.	[39]
			Scientists can be trusted because they adhere to strict rules and standards in their work.	
			Scientists can be trusted because they work for the common good.	
			Scientists can be trusted because they inform the public about the relevant results of their research.	
			Scientists can be trusted because they sufficiently involve the public in their research.	
Affective trust in new food ^a^	4	0.76	I am constantly sampling new and different foods.	[42]
			I like food from different cultures.	
			I will try new foods at dinner parties.	
			I will eat almost anything.	
Dispositional trust in sources of food information ^b^	7	0.81	The government (e.g., FDA, USDA, etc.)	[46]
			Food processors/manufacturers (e.g., retailers)	
			Grocery stores	
			News media	
			Social media	
			Advocacy organizations	
			Health professionals (doctor, nurse, dietitian)	

Note: ^a^ scale used 1 = Strongly Disagree; 2 = Disagree; 3 = Neither Agree nor Disagree; 4 = Agree; 5 = Strongly Agree; ^b^ scale used 1 = Very Untrustworthy; 2 = Somewhat Untrustworthy; 3 = Neither Trustworthy nor Untrustworthy; 4 = Somewhat Trustworthy; 5 = Very Trustworthy.

**Table 2:** Demographics of respondents prior to weighting (*N* = 1011).

	N	%
Sex		
Male	465	46.0
Female	546	54.0
Race *		
White	757	74.9
Black	149	14.7
Asian	71	7.0
American Indian or Alaska Native	64	6.3
Other	45	4.5
Hispanic ethnicity	190	18.8
Education		
Less than 12th grade	27	2.7
High school diploma	252	24.9
Some college	241	23.8
2-year college degree	127	12.6
4-year college degree	230	22.7
Graduate or Professional degree	134	13.3
Marital Status		
Single	333	32.9
Married	363	35.9
Living with a partner, not married	85	8.4
Divorced	148	14.6
Separated	17	1.7
Widowed	65	6.4
Family Income		
Less than USD 24,999	238	23.5
USD 25,000–49,999	283	28.0
USD 50,000–74,999	208	20.6
USD 75,000–149,999	207	20.5
USD 150,000–249,999	51	5.0
USD 250,000 or more	24	2.4
Children under the age of 18 currently living in the home		
0	725	71.7
1	147	14.5
2	103	10.2
3 or more	36	3.6
Special diet		
Vegetarian (no meat, chicken, or fish/seafood)	54	5.4
Pescatarian (no flesh of any animal except fish/seafood)	21	2.1
Vegan (no animal or seafood products of any kind, including dairy)	18	1.8
Paleo (no dairy or grain products and no processed food)	20	2.0
Political ideology		
Very liberal	126	12.5
Liberal	186	18.4
Moderate	440	43.5
Conservative	160	15.8
Very conservative	99	9.8

Note: * respondents allowed to select more than one race; therefore, percentages do not equal 100%.

**Table 3:** Respondents’ levels of cognitive, affective, and dispositional trust and age based on segments.

	Lack Trust *n* = 108 *M (SD)*	Trusting *n* = 174 *M (SD)*	On the Fence *n* = 272 *M (SD)*	Trust New Food Not Science or Sources *n* = 202 *M (SD)*	Trust Science Not New Food *n* = 255 *M (SD)*	F
Cognitive trust in science	2.73 (0.73) ^e^	4.51 (0.38) ^a^	3.76 (0.44) ^c^	3.08 (0.56) ^d^	3.91 (0.50) ^b^	298.96 *
Affective trust in new food	2.23 (0.53) ^e^	4.21 (0.54) ^a^	4.01 (0.41) ^b^	3.66 (0.52) ^c^	2.71 (0.51) ^d^	499.59 *
Dispositional trust in sources of food info.	2.64 (0.49) ^c^	4.09 (0.49) ^a^	3.32 (0.43) ^b^	2.47 (0.44) ^c^	3.47 (0.53) ^b^	335.51 *
Age	56.57 (17.55)	46.26 (16.97)	49.35 (17.65)	50.63 (17.96)	56.64 (18.04)	12.53 *

Note: * *p* < 0.001. Superscript letters denote statistically significant differences across clusters.

**Table 4:** Demographic differences based on trust segment alignment.

	Lack Trust *n* = 108 %	Trusting *n* = 174 %	On the Fence *n* = 272 %	Trust New Food Not Science or Sources *n* = 202 %	Trust Science Not New Food *n* = 255 %	X^2^	Cramer’s V
Sex						17.29 *	0.13
Male	35.2 ^2^	49.4	50.4	52.5 ^1^	38.4 ^2^		
Female	64.8 ^1^	50.6	49.6	47.5 ^2^	61.6 ^1^		
Race						N/A	
White	69.7	73.5	69.5	70.7	66.4		
Black	12.6	12.4	12.5	7.7	21.5		
Asian	5.0	7.0	6.1	8.6	5.7		
American Indian or Alaska Native	6.7	4.9	6.4	9.0	3.0		
Other	5.9	2.2	5.4	4.1	3.4		
Hispanic ethnicity	11.1	25.3	21.0	16.3	17.3	11.01 *	0.10
Education						30.98 ^t^	0.09
Less than 12th grade	3.7	2.9	1.1	3.0	3.5		
High school diploma	26.9	20.7	22.1	24.3	30.6 ^1^		
Some college	24.1	22.4	24.6	25.7	22.4		
2-year college degree	15.7	8.0 ^2^	14.3	11.9	12.9		
4-year college degree	21.3	25.3	21.7	24.3	21.6		
Graduate or professional degree	8.3	20.7 ^1^	16.2	10.9	9.0 ^2^		
Marital status						37.27 *	0.10
Single	29.6	37.4	33.1	31.7	32.2		
Married	30.6	34.5	37.9	42.6 ^1^	31.8		
Living with a partner, not married	9.3	9.8	6.6	8.9	8.6		
Divorced	13.9	13.8	14.7	10.4	18.8 ^1^		
Separated	2.8	1.1	3.3 ^1^	1.0	0.4		
Widowed	13.9 ^1^	3.4	4.4	5.4	8.2		
Family income						47.25 **	0.11
Less than USD 24,999	31.5 ^1^	17.8	19.9	21.3	29.8 ^1^		
USD 25,000–49,999	27.8	24.7	28.3	26.7	31.0		
USD 50,000–74,999	19.4	13.8 ^2^	24.6 ^1^	25.2 ^1^	17.6		
USD 75,000–149,999	15.7	29.9 ^1^	20.2	21.3	15.7 ^2^		
USD 150,000–249,999	3.7	9.8 ^1^	5.1	3.0	3.9		
USD 250,000 or more	1.9	4.0	1.8	2.5	2.0		
Children under the age of 18 currently living in the home						40.58 **	0.10
0	80.6 ^1^	59.2 ^2^	69.1	71.3	79.6 ^1^		
1	10.2	21.8 ^1^	16.5	12.4	11.0		
2	3.7 ^2^	15.5 ^1^	11.4	12.4 ^1^	6.3 ^2^		
3 or more	5.5	3.5	3.0	4.0	3.2		
Special diet						34.45	n/a
Vegetarian (no meat, chicken, or fish/seafood)	5.6	2.9	2.2	1.5	6.3		
Pescatarian (no flesh of any animal except fish/seafood)	1.9	1.7	0.7	0.0	0.0		
Vegan (no animal or seafood products of any kind, including dairy)	2.8	0.6	0.4	1.0	1.6		
Paleo (no dairy or grain products and no processed food)	1.9	2.9	0.7	2.5	2.7		
Political ideology						105.37 **	0.16
Very liberal	6.5	28.7 ^1^	10.3	6.4 ^2^	11.0		
Liberal	9.3 ^2^	19.5	21.7	12.9 ^2^	22.4 ^1^		
Moderate	41.7	37.9	44.5	44.6	46.3		
Conservative	23.1 ^1^	6.9 ^2^	18.0	19.8	13.3		
Very conservative	19.4 ^1^	6.9	5.5 ^2^	16.3 ^1^	7.1		

Note: ^t^ *p* < 0.05; * *p* < 0.01; ** *p* < 0.001; ^1^ adjusted residual > |1.96|, indicating significant differences beyond expected under independence (*p* < 0.05), more than expected; ^2^ adjusted residual > |1.96|, indicating significant differences beyond expected under independence (*p* < 0.05), less than expected.

## Data Availability

The datasets presented in this article are not readily available because the data are part of an ongoing study. Requests to access the datasets should be directed to the corresponding author.
